# Variation in synonymous codon usage in Paenibacillus sp. 32O-W genome

**DOI:** 10.6026/97320630012396

**Published:** 2016-12-01

**Authors:** Sushanta Deb, Surajit Basak

**Affiliations:** 1 Department of Molecular Biology & Bioinformatics, Tripura University, Suryamaninagar, Tripura-799022, India; 2 Bioinformatics Centre, Tripura University, Suryamaninagar, Tripura-799022, India

**Keywords:** Variation, codon usage, Paenibacillus sp. 320-W genome

## Abstract

Paenibacillus sp. 32O-W, which is attributed for biodesulfurization of petroleum, has 56.34% genomic G+C content. Correspondence analysis on Relative Synonymous Codon Usage (RSCU) of the Paenibacillus sp. 32O-W genome has revealed the two different trends of codon usage variation. Two sets of genes have been identified representing the two distinct pattern of codon usage in this bacterial genome. We have measured several codon usage indices to understand the influencing factors governing the differential pattern of codon usage variation in this bacterial genome. We also observed significant differences in many protein properties between the two gene sets (e.g., hydrophobicity, protein biosynthetic cost, protein aggregation propensity). The compositional difference between the two sets of genes and the difference in their potential gene expressivity are the driving force for the differences in protein biosynthetic cost and aggregation propensity. Based on our results we argue that codon usage variation in Paenibacillus sp. 32O-W genome is actually influenced by both mutational bias and translational selection.

## Background

Though Paenibacillus sp. 32O-W, cannot metabolize derivatives of dibenzothiophene but surprisingly together with Paenibacillus napthalenovorans 32O-Y, able to metabolize derivatives of dibenzothiophene even in a advanced speed than that of Paenibacillus napthalenovorans 32O-Y alone [1]. Research on synonymous codon usage gives the information about the molecular evolution of individual gene, data obtained from the research are being utilized to develop algorithms for gene recognition, to design DNA primers and discern the events of Horizontal gene transfer [2]. Several earlier studies suggested those different varieties of factors contributing to the biased usage of synonymous codons such as gene length, proteins secondary structure and gene density, CpG islands, gene expression level and other things. To date studies revealed that the two major phenomena determine the codon usage was mutational bias or natural selection there is no any unified theory describing codon usage. It has been established that within genomes, highly expressed genes are encoded by preferred synonymous codon very often than other, less highly expressed genes and preferred codon are those that tend to match the more abundant anticodon. Studies of codon usage patterns on genome have open the aspects to understand the basic features of the molecular organization in genomes. Varying strength of selection acting on evolutionarily conserved amino acid residues exhibits stronger bias. In contrast, weaker codon usage bias observed in evolutionary variable residues [3,4].

Studies have been reported that there is a negative association between codon usage bias and average biosynthetic cost of the amino acids incorporated into the expressed protein [5]. The amino acid having high biosynthetic cost has the propensity to be less encoded by the genes with greater codon usage bias in contrast lowly biased genes incorporated the amino acid with high biosynthetic cost [6]. Through the course of evolution prokaryotic cells adapted use less energetically costly amino acids in highly expressed proteins and provide an insight about the connection of cellular metabolism and the evolution of its genome sequence.

The phenomenon of protein misfolding is also associated with the expression of the proteins in the cell [7]. The evolution of protein sequence might have influenced by their respective aggregation propensity. In a protein sequence the aggregation prone regions are typically encoded by hydrophobic amino acids (valine, leucine isoleucine and phenylalanine) [7]. Organisms with AT biased genome have smaller efficiency of protein folding and A+T biased mutation at the DNA level drives the translated product into more hydrophobic [8]. To better understand the genetic features of Paenibacillus sp. 32O-W multiple factors influencing synonymous codon usage patterns in Paenibacillus sp. 32O-W were analyzed in this study.

## Methodology

### Gene sequences

Complete coding sequences (CDS) of Paenibacillus sp. 32O-W genome were retrieved from Gene bank (CP013653). To minimize the sampling errors, CDS with more than 300 nucleotides were chosen for analysis with correct start and stop codons in every CDS [9].

### Indices of codon usage

The extent of codon bias of an individual gene were measured by obtaining the values of effective number of codon (NC) providing the values ranging from 20 for the gene with extreme bias using only one codon per amino acid, to 61 for a gene using all the codons allotted for each amino acid randomly with no bias. The enc values obtained by using the Codon W software. The extent of biasness of the preferred codon in highly expressed genes was estimated using the codon adaptation Index.CAI value ranges between 0 to 1,higher value indicate the higher codon usage bias with higher expression level, this indices were calculated using codon w [10].

GRAVY score or General Average Hydropathicity of a hypothetical translated gene product is known as Hydropathicity value. It is calculated as the arithmetic mean of the sum of the hydropathic indices of each amino acid. GRAVY (General Average Hydropathicity) values are calculated as arithmetic mean of the hydropathy values of all the amino acids in the gene product. The Hydrophilic protein having more negative gravy value in contrast hydrophobic protein showing more positive gravy value [11].

### COA (correspondence analysis)

The most widely accepted method for multivariate statistical analysis to study the codon usage pattern is correspondence analysis (COA) [12]. Since there are a total of 59 synonymous codons excluding Met, Trp, and termination codons, partitioning the variation along 59 orthogonal axis, with 41 degree of freedom. This analysis identifies the axes, which represent the most prominent factors contributing to the variation among genes.

### Software used

The program codonW 4.1 were used to measure the indices of codon usage .The statistical analysis was performed using SPSS 16 for windows. Software package DAMBE were used to obtain the values of amino acid biosynthetic cost for each translated gene product and using the program TANGO [13] protein aggregation score were determined.

## Results and Discussion

**Figure 1:**
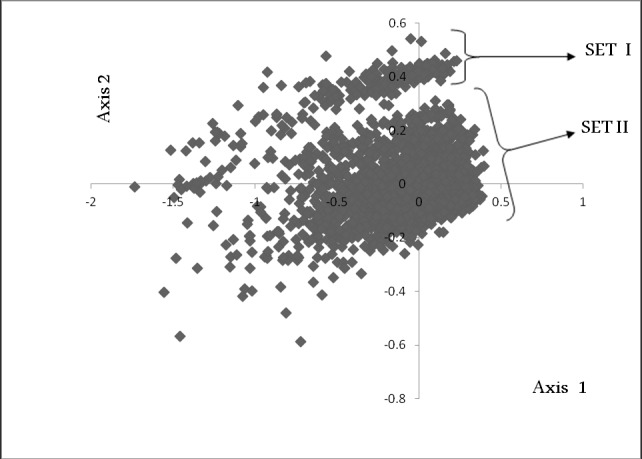
Distinct codon usage pattern among the genes of Paenibacillus sp. 32O-W genome named as SET I and SET II gene sets

Several studies on codon usage have established that a considerable heterogeneity prevails among genes of the same species [14, 15,16,17]. The genome of Paenibacillus sp. 32O-W bacteria exhibits an unusual codon usage trend among the genes. We have performed a correspondence analysis (COA) on RSCU, which indicates that there are two gene sets with distinct codon usage pattern. These two gene sets with different codon usage pattern are clustered separately on Axis1 (horizontal axis) and are referred to as SET I and SET II (Figure 1). SET I cluster contains 241 genes and SET II cluster contains 4512 genes. Distinctive codon usage pattern between these two sets of genes of the same genome might be the result of a combination of several influencing factors [18, 19,20, 21,22, 23,24]. To study the factors governing the distinct codon usage pattern among the genes of Paenibacillus sp. 32O-W genome we have measured the hydrophobicity score of the proteins encoded by the Paenibacillus sp. 32O-W genome. We found that the total hydrophobicity score of the two sets of genes are significantly different (P<0.01) with higher hydrophobicity in SET I genes. It was observed that protein hydrophobicity exhibits a negative correlation with genomic GC content [25]. We observed that average GC content of SET I genes (55%) is lower than the SET II genes (56.61%) in this bacterial genome. We were interested to see if this compositional constraint (i.e lower GC content of the SET I genes) influences the hydrophobicity of the gene product. As the SET I genes showing higher hydrophobicity value than SET II genes, we compared the Relative Amino acid Usage (RAAU) values of the hydrophobic amino acids of SET I and SET II. Two hydrophobic amino acids (Leucine and Isoleucine) show statistically significant difference (P < 0.01) in their RAAU values between these two gene sets. The average RAAU value of both Isoleucine and Ieucine is higher in SET I genes compared to SET II genes.

Isoleucine and Leucine are encoded by three and six synonymous codons respectively. We have calculated the Relative Synonymous Codon Usage (RSCU) values for the synonymous codons of Isoleucine and Leucine (Table 1). For Isoleucine synonymous codons, the RSCU values of ATT and ATA are significantly higher in SET I than in SET II; ATC does not have any significant difference in RSCU between the two sets. For Leucine synonymous codons, the RSCU values of TTA, CTT, CTA are significantly higher in SET I than in SET II. We did not observe any significant difference in RSCU values of TTG, CTC, and CTG between the two sets. A mutational bias towards using AT-ending codons to encode hydrophobic amino acids is quite prominent in the above observation. It implies that the compositional constraint on codon usage is actually influencing the variation in hydrophobicity of both the gene set of the whole genome of Paenibacillus sp. 32O-W.

Previous studies established that amino acid with lower biosynthetic cost preferably found in protein product of highly expressed gene, in contrast lowly expressed gene product tends to favor amino acid with higher average biosynthetic cost [26]. We observed that the biosynthetic cost of SET I and SET II genes were significantly different (P<0.05) in this bacterial genome with higher amino acid biosynthetic cost in SET I genes. This increased amino acid biosynthetic cost in the SET I genes predicts that SET I gene might be lowly expressed, which may be a causal factor for the distinct codon usage pattern of SET I genes from SET II genes. We have used Codon Adaptation Index (CAI) as the potential measure of gene expression. The CAI value of SET I genes were found to be lower than that of the SET II genes suggesting that the potential expression level of SET I genes are low in this bacterial genome, which in turn supports the view that codon usage of highly expressed genes tends to avoid AT richness in their codon [27].

It is reported that gene expression level is highly correlated with solubility of the encoded protein. Highly translated proteins intended to be more soluble than the proteins with low expression rates. Protein aggregation results in unfavorable condition for the cell, such as reduced amino acid recycling, recruitment and blockage of molecular chaperones and proteases, formation of toxic polypeptides or simply the loss of function of the misfolded protein [28,29]. Protein aggregation also has the beneficial aspect; protein aggregates contribute to exceptional stability, compactness and forms of organization that could not be achieved by monomeric or oligomeric conformations [7]. Several earlier studies reported that protein aggregation in organisms is beneficial for the adaptation in the diverse environment [30, 31,32]. Considering the entire phenomenon due to aggregation property of protein we have predicted the protein aggregation score for all the genes of both the gene sets using a statistical mechanics algorithm, TANGO. Relative Aggregation Propensity (RAP) was obtained using the aggregation score derived from the TANGO program. To evaluate whether the propensity to form aggregation by the gene product of this two gene sets varies significantly, we performed a statistical test and found a significant difference (P < 0.05) of aggregation score between the two gene sets. Protein aggregation tendency were higher in SET I genes, this might be due to the higher hydrophobicity of the SET I genes.

In the present study, significant compositional difference is found between the two sets of genes with AT rich genes in the SET I gene set. The proteins encoded by SET I genes set are hydrophobic in nature and this may drive the AT richness in this group of genes. The genes in the SET I display AT mutational bias with low amino acid biosynthetic cost having lower gene expression level. Translational selection pressure may hardly influencing the SET I gene set, aggregation propensity is also higher in this gene set. This study supports that lowly expressed gene have higher aggregation propensity. The factor that is hydrophobicity, amino acid biosynthetic cost, expression level and aggregation propensity playing a significant role for the distinct codon usage of SET I genes from the other genes (SET II genes) of the bacterial genome of Paenibacillus sp. 32O-W.

## Conclusion

Different factors affecting codon usage bias in of the Paenibacillus sp. 32O-W genome has been analysed in the present study. Natural selection is known to play an important role in shaping the codon usage of an organism. Though codon usage of SET I and SET II genes of Paenibacillus sp. 32O-W is mainly governed by compositional constraint, natural selection (translational selection) is also contributing in shaping the codon usage variation between these two gene sets in this bacterial genome. It is also worth to note that hydrophobicity of gene product also appears as a major factor for distinct codon usage pattern in SET I and SET II genes of this bacterial genome.

## Figures and Tables

**Table 1:** RSCU values of Leucine and Isolucine between SET I and SET II genes (‘*’ indicates signifance at p<0.1 and NS= Non Significant)

Amino Acid	Codons	RSCU of SET I	RSCU of SET II	Statistical significance
Leucine	TTA	0.24	0.18	*
	TTG	1.29	1.26	NS
	CTT	0.82	0.72	*
	CTC	1.04	1.13	NS
	CTA	1.02	0.96	*
	CTG	0.68	0.7	NS
Isoleucine	ATT	1.07	1.02	*
	ATC	2.76	2.78	NS
	ATA	0.27	0.24	*

**Table 2:** Molecular interaction of carborane substituted Withaferin A compound derivatives with IDO

Derivative no	No of H bonds	H-bonds forming residues	Hydrophobic (Green)	Polar (Sky blue)	Charged (-ve) Red	Charged (+ve) Purple	Glycine (Yellow)
1	11	Gly265, Gln266, Gly261, Gly236, Arg297, Tyr298	Tyr298	Gln266	--	Arg297	Gly265, Gly261, Gly236
2	4	Lys238, Arg231, Ser235, Ala264	Ala264	Ser235	--	Lys238, Arg231	--
3	6	Leu234, Arg231, Lys238, Asp294, Tyr298	Leu234, Tyr298	--	Asp294,	Arg231, Lys238	--
4	--	--	--	--	--	--	--
5	4	Ser234, Gln266, Ala264	Ala264	Ser234, Gln266	--	--	--
6	4	Gly261, Gly236, Lys238, Asn240	--	Asn240	--	Lys238	Gly261, Gly236
7	4	Leu234, Lys238, Asn240	Leu234	Asn240	--	Lys238	
8	10	Phe259, Lys238, Asn240, Gln242, Gly261, Gly236, Trp92, Arg231, Ser235	Phe259, Trp92	Asn240, Gln242, Ser235	--	Lys238, Arg231	Gly261, Gly236
9	4	Arg231, Ser235, Gly261	--	Ser235	--	Arg231	Gly261
10	12	Phe259, Ser235, Lys238, Asn240, Arg231, Gly236, Gly261	Phe259	Ser235, Asn240	--	Lys238, Arg231	Gly236, Gly261

**Table 3:** MD simulation statistics for the compound 01 in complex with IDO

MD run	Total energy (Kcal/mol)		Intra H-Bond		Inter H-Bonds		RMSD		ROG	
Range	Mean	Range	Mean	Range	Mean	Range	Mean	Range	Mean
Run 1	-9574 to -8136	-8832	251 to 307	279	0 to 5	1	0.0 to 3.0	2.2	21.3 to 22.1	21.8
Run 2	-9516 to -7948	-8643	255 to 318	287	0 to 4	1	0.0 to 2.8	2.1	21.4 to 21.9	21.6
